# The return to work experiences of middle-aged Australian workers diagnosed with colorectal cancer: a matched cohort study

**DOI:** 10.1186/1471-2458-14-963

**Published:** 2014-09-17

**Authors:** Louisa G Gordon, Vanessa L Beesley, Brigid M Lynch, Gabor Mihala, Catherine McGrath, Nicholas Graves, Penelope M Webb

**Affiliations:** Griffith Health Institute, Centre for Applied Health Economics, Griffith University, University Drive, Meadowbrook, QLD 4131 Brisbane, Australia; QIMR Berghofer Medical Research Institute, Royal Brisbane Hospital, Locked Bag 2000, QLD 4029 Brisbane, Australia; School of Public Health, Queensland University of Technology, Victoria Park Rd, Kelvin Grove, Brisbane, QLD 4006 Australia; Physical Activity Laboratory, Baker IDI Heart and Diabetes Institute, 75 Commercial Rd, Melbourne, VIC 3004 Australia; Melbourne School of Population and Global Health, Faculty of Medicine, Dentistry and Health Sciences, The University of Melbourne, Melbourne, VIC 3010 Australia

**Keywords:** Colorectal cancer, Return to work, Employment outcomes, Middle-aged

## Abstract

**Background:**

Few studies have been undertaken to understand the employment impact in patients with colorectal cancer and none in middle-aged individuals with cancer. This study described transitions in, and key factors influencing, work participation during the 12 months following a diagnosis of colorectal cancer.

**Methods:**

We enrolled 239 adults during 2010 and 2011who were employed at the time of their colorectal cancer diagnosis and were prospectively followed over 12 months. They were compared to an age- and gender-matched general population group of 717 adults from the Household, Income and Labour Dynamics in Australia (HILDA) Survey. Data were collected using telephone and postal surveys. Primary outcomes included work participation at 12 months, changes in hours worked and time to work re-entry. Multivariable logistic and Cox proportional hazards models were undertaken.

**Results:**

A significantly higher proportion of participants with colorectal cancer (27%) had stopped working at 12 months than participants from the comparison group (8%) (p < 0.001). Participants with cancer who returned to work took a median of 91 days off work (25–75 percentiles: 14–183 days). For participants with cancer, predictors of not working at 12 months included: being older, lower BMI and lower physical well-being. Factors related to delayed work re-entry included not being university-educated, working for an employer with more than 20 employees in a non-professional or managerial role, longer hospital stay, poorer perceived financial status and having or had chemotherapy.

**Conclusions:**

In middle-adulthood, those working and diagnosed with colorectal cancer can expect to take around three months off work. Individuals treated with chemotherapy, without a university degree and from large employers could be targeted for specific assistance for a more timely work entry.

**Trial registration:**

ACTRN12611000530921

**Electronic supplementary material:**

The online version of this article (doi:10.1186/1471-2458-14-963) contains supplementary material, which is available to authorized users.

## Background

Each year in Australia over 64,000 cancers, or 56% of all cancers, are diagnosed in adults of working ages (20–64 years) [1], a proportion which is broadly similar to other developed countries. Cancer treatments often involve prolonged periods of adjuvant therapy and accompanying side-effects and thus disrupt an individual’s employment, earnings and other role activities. Although not all patients wish to return to work, resumption of work is often seen as a positive step in a cancer survivor’s recovery and generally signifies a milestone on the patient’s physical and mental return to their normal activities. A cancer experience that causes major disruption in the work role can become a source of high distress and adversely affect health-related quality of life (HRQoL) and economic security [2].

Colorectal cancer is the most common cancer in men and women combined in most Western nations and increased numbers are expected due to ageing populations [3]. An experience of colorectal cancer may cause individuals to have poorer employment outcomes than for other cancer types (e.g., breast, prostate, skin, lymphoma) due to the disease-specific issues that may impact the ability to perform work [4], such as altered bowel habits or managing an intestinal stoma. In addition, adjuvant chemotherapy is given to approximately 50% of patients with colorectal cancer [5, 6] and during receipt of this therapy an individual’s work ability can be impeded through muscle fatigue, anaemia, hair loss, medication effects, peripheral neuropathy and impaired mental acuity or ‘chemo brain’ [7]. Many chemotherapies also have immunosuppressive properties which may require patients receiving them to avoid crowds or indoor workplaces [7].

In a recent review of 64 studies that specifically addressed employment issues following cancer, pooled results indicated 64% of those working at the time of diagnosis returned to work after cancer (range 24-94% in individual studies) [8]. Of those who returned to work, 40% had continued to work through their treatment or had returned to work by 6 months, 62% were working at 12 months, 73% by 18 months and 89% by 24 months [9]. Among individuals with different cancer types, determinants of delayed return to, or stopping work include: older age [10–12]; physically-demanding work [10–13]; being female [9–11, 13]; presence of comorbidities [14]; being married [9, 11]; fatigue [14]; lower education [11, 15]; and treatment with chemotherapy [9]. Most of the research to date has concentrated on breast cancer survivors so the relevance for a colorectal cancer population is unclear [8].

This study reports the results of a prospective, population-based study of middle-aged Australian adults with colorectal cancer and their work experiences. We have looked at employment outcomes specifically targeting individuals aged 45 to 64 years who were still in the workforce and no other study has done this for any cancer population. We chose these individuals because they will most likely be at a similar stage of life (middle adulthood) and therefore not planning immediate retirement but rather, many would be in the midst of their careers, with many in senior and managerial roles. In addition, unlike most studies that focus on medical or socio-demographic factors as predictors of work return, our study included many work-related factors that could influence return to work such as: type of work, work schedule (e.g., regular daytime, shift-work), type of employer, workplace size, sick leave provisions, degree of work autonomy and the level of support from employers and colleagues. We excluded those aged less than 45 years because colorectal cancer is rare in individuals less than 45 and these individuals may be less engaged with the workforce if they have parenting commitments to young children. The key aims of the study were to 1) describe *transitions* in work participation over a 12-month period following a primary diagnosis of colorectal cancer compared to individuals without cancer; 2) identify the key factors influencing *work participation* during or after cancer treatment compared to individuals without cancer; and 3) identify the key factors influencing *time to work re-entry* after cancer treatment among individuals taking work leave as a result of their cancer.

## Methods

### Study participants

Full details of the study have been previously described [16]. In brief, a prospective, population-based study enrolled middle-aged (45–64 years) men and women who were in the paid workforce and newly-diagnosed with colorectal cancer. Participants resided in Queensland, Australia, were able to complete questions in English, were enrolled through the Queensland Cancer Registry between January 2010 and September 2011 and had a histologically-confirmed diagnosis of colorectal cancer. Participants were followed for 12 months. These participants were matched by gender and 5-year age group to data from a nationally representative sample of comparison individuals in the paid workforce who were randomly selected from those who participated in both Waves 10 (2010) and 11 (2011) of the Household, Income and Labour Dynamics in Australia (HILDA) Survey dataset [17].

Underpinning the study design was the theoretical model by Steiner *et al* who proposed a social approach to work outcomes for cancer survivors where the relationship between medical/patient factors and work resumption are moderated by the individual’s work environment and socio-demographic factors [18]. Ethics approval for the study was obtained from the Human Ethics Research Committees of QIMR Berghofer Medical Research Institute (P1128), Griffith University (MED/19/09/HREC) and the Queensland Health Research Ethics and Governance Unit (RD003482).

### Data collection and instruments

#### Cancer group

Data were collected from pathology reports on tumor site (colon, rectum), histopathological tumor type, degree of differentiation or grade, degree of metastasis and stage of disease (i.e., American Joint Committee of Cancer (AJCC) and Dukes staging). Participants completed structured telephone interviews at 6 and 12 months after diagnosis. At the 6-month data collection time point, participants recalled work-related information at the time they were diagnosed with cancer (baseline) while at 12 months they recalled work information since the 6-month data collection. Interviews were conducted by trained and experienced interviewers. To supplement the data collected by telephone interview, and to address items of a more sensitive nature (i.e., health behaviours, HRQoL, financial strain), participants completed postal surveys sent immediately after both telephone interviews.

#### General population comparison group

Baseline data for the general population group were taken from the Wave 10 interview (conducted during 2010) and 12 month data from Wave 11 (conducted during 2011). The HILDA survey data were collected through face-to-face interviews.

Generic baseline socio-demographic and work status information were obtained for both the cancer and general population groups using identical items. Work status items included questions from validated tools from Australian government surveys (e.g., Australian Bureau of Statistics (ABS) Labour Force Survey, ABS 1999 Survey of Living Standards) [19] and included: employment status, usual and preferred weekly hours, reasons for working part-time, occupation, occupation change, industry, paid leave provisions, employer type, workplace size, work autonomy, perceived financial status. Work transitions were categorised as four mutually exclusive change categories from baseline to 12 months: ‘retired/ceased work’ (changed to 0 hours work), ‘increased work’ (by ≥4 hours per week), ‘decreased work’ (by ≥4 hours per week) and ‘unchanged’ (work hours changed by <4 hours per week). A four-hour margin in work transition categories was chosen to focus on meaningful changes in hours worked. The study was statistically powered to detect a 15% difference in the proportions of work participation across the cancer and general population groups [16].

### Analyses

Baseline socio-demographic and cancer pathology data were compared for the participants and non-participants, and between the cancer and general population groups, using chi-square, Fisher’s exact and t-tests for statistically significant differences. Descriptive analyses charted employment transitions from diagnosis to 6- and 12-months among the cancer group and over 12-months (i.e., the 2010–2011 year period) among the general population group. The extent of missing data and drop out in the cancer group was analysed by examining baseline variables for the ‘completers’ and ‘non-completers’ to identify possible biases.

Multivariable logistic regression modelling with backward removal of variables identified explanatory variables (i.e., socio-demographic, clinical and work status variables outlines above) that were significantly associated with work participation in the cancer and general population groups. Cox proportional hazards modelling with backward removal of variables assessed the hazard of work re-entry (where a hazard ratio of <1 is associated with a longer time to work resumption) in participants with cancer. Where a person worked continually through their treatment, we assumed one day was taken off work to enable these individuals to have an ‘event’ (work resumption), and be captured in the returned workers category. The number of days off work was entered as the time variable in the model, and those permanently retired were excluded from the analysis. Analyses were stratified by gender but as there negligible differences the data were subsequently pooled. The proportional-hazards assumption was checked for the multivariable model. Multiple imputation was employed to handle missing data where the proportion of missing values was between 5 and 30% and the number of imputations was equal to or greater than the percentage of incomplete cases [20]. STATA SE (Version 12.1) was used for all analyses. Statistical significance was at the p < 0.05 level.

## Results

### Response rates among the cancer group

**Figure 1:**
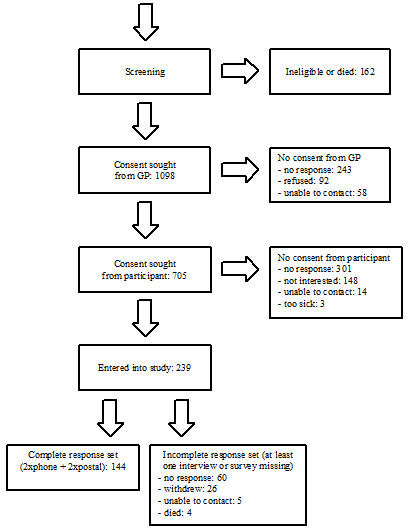
**Participant flow through the study for the cancer group.**

A total of 1,260 men and women diagnosed with colorectal cancer during the study period and aged 45–64 were identified as potentially eligible for the study. Of these, 162 were subsequently found to be as ineligible (i.e., not in paid employment or outside age-group) or had died. For 393 individuals, doctor’s consent was not received to enable the researchers to contact potential participants, and a further 466 individuals did not provide consent. A lack of response to follow-up letters and telephone calls was the main reason for non-consent from both the doctors and potential participants. In total, 239 men and women were confirmed as eligible and consented to the study (Figure 1), giving a recruitment rate of 239/1,098 (22%) and a participant response rate of 239/705 (34%).

### Comparison of cancer group participants and non-participants

A comparison of the participants and non-participants showed no statistically significant differences for place of residence, diagnosis by excision or biopsy, cancer site, histology, cancer grade and AJCC stage. However, a significantly higher proportion of participants were male (n = 159, 67%) versus non-participants (n = 500, 58%) (p = 0.02). Participants were also slightly younger and a lower proportion had advanced cancer (Additional file 1: Table S1).

### Comparison of cancer group completers and non-completers

Analyses comparing the ‘completers’ and ‘non-completers’ of the telephone interviews revealed no statistically significant differences in any socio-demographic, cancer or health characteristics (Additional file 1: Table S2). There was a similar finding for ‘completers’ and ‘non-completers’ of the postal surveys, with the exception of fewer surveys being returned by those living in major cities among the ‘non-completers’ (44% vs 51%, p = 0.03).

### Baseline characteristics of the cancer group

The mean age of the cancer group was 56 years (SD 5.5), 67% were male, 49% lived in a major city, 81% were married or partnered and 22% were university educated (Table 1). In the 136 participants where AJCC stage was recorded, 46 (34%) had cancer stage I, 40 (29%) had stage II, 47 (35%) had stage III and 3 (2%) had stage IV. Of the 214 participants that had surgery for their primary treatment, 129 (60%) participants underwent laparoscopic surgery, while 64 (30%) participants were fitted with a stoma (13 with permanent stomas). Forty participants (19%) experienced complications during their surgical hospital stay, and the median length of stay was six days (25–75 percentiles: 4–9 days). In terms of adjuvant therapy, 54% had chemotherapy and 16% had radiotherapy. Twenty-six participants (20%) reported they were hospitalised for side-effects attributed to their adjuvant therapy.

**Table 1:** **Baseline characteristics in the colorectal cancer and general population groups**

		Cancer	General population	*p* ^*1*^
		N = 239	N = 717	
Gender	Male	160 (67%)	480 (67%)	1.00
Age	45-49 years	35 (15%)	105 (15%)	1.00
	50-54 years	51 (21%)	153 (21%)	
	55-59 years	74 (31%)	222 (31%)	
	60-64 years	79 (33%)	237 (33%)	
Indigenous	Yes	3 (1%)	10 (2%)	0.57
Country of birth	Australia	180 (81%)	511 (71%)	0.01
	Other English-speaking	25 (11%)	112 (16%)	
	Non-English speaking	17 (8%)	93 (13%)	
Rurality	Major city	117(49%)	445 (62%)	<0.01
	Inner regional	66 (28%)	169 (24%)	
	Outer regional	44 (18%)	85 (12%)	
	Remote	8 (3%)	13 (2%)	
	Very remote	4 (2%)	4 (1%)	
Marital status	Single	22 (10%)	38 (5%)	<0.01
	Widowed	2 (1%)	18 (3%)	
	Divorced/separated	19 (9%)	128 (18%)	
	Married/partnered	179 (81%)	532 (74%)	
Highest level of education	No formal schooling	1 (1%)	0 (0%)	<0.01
	Primary school	17 (8%)	23 (3%)	
	Junior high school	90 (41%)	152 (21%)	
	Senior high school	36 (16%)	72 (10%)	
	Trade/technical/diploma	29 (13%)	254 (36%)	
	University	48 (22%)	215 (30%)	
Other co-morbidities	Arthritis	40 (18%)	135 (19%)	0.67
	Asthma	20 (9%)	53 (8%)	0.49
	Cancer (other than colorectal)	29 (13%)	18 (2%)	<0.01
	Bronchitis/emphysema	11 (5%)	14 (2%)	0.02
	Type 2 diabetes	24 (10%)	38 (5%)	<0.01
	Depression/anxiety	20 (9%)	49 (7%)	0.32
	Heart/coronary disease	15 (7%)	25 (4%)	0.04
	High blood pressure	56 (25%)	164 (24%)	0.57
	Other circ. condition	7 (3%)	8 (1%)	0.04
Household income gross annual in 2010 AUD	Less than $ 36400	16 (8%)	85 (12%)	<0.01
	$ 36400 - $ 77999	54 (28%)	186 (27%)	
	$ 78000 - $ 103999	47 (24%)	100 (14%)	
	$ 104000 - above	76 (39%)	326 (47%)	
Occupation type	Professional/managerial	54 (25%)	303 (42%)	<0.01
	Skilled clerical/sales	98 (45%)	159 (26%)	
	Skilled trades/production	64 (30%)	223 (31%)	
Work status	Full-time (≥32 hours/week)	170 (78%)	526 (76%)	0.54
	Part-time (<32 hours/week)	47 (22%)	163 (24%)	
Time (years) worked for current employer	Median (25,75%)	8 (3.0,17.0)	10 (3.0,20.0)	0.57^2^
Work schedule	Regular daytime	160 (74%)	542 (76%)	0.58
	Other	57 (26%)	175 (24%)	
Contract type	Fixed	17 (8%)	46 (9%)	0.61
	Casual	26 (12%)	78 (15%)	
	Permanent	173 (80%)	412 (77%)	

^1^calculated using chi-squared test; frequencies and proportions displayed, unless otherwise indicated; sums of frequencies can be less than the overall group sizes due to missing data.

^2^t-test.

### Comparison of the cancer and general population group

There were several differences in the baseline characteristics between the cancer and general population group participants (Table 1) after matching by gender and 5-year age-group. Participants with cancer were significantly more likely to be Australian born, less likely to be widowed or divorced/separated, had lower education and income levels, and more likely to have other comorbidity, particularly diabetes or another cancer (Table 1).

### Employment changes

At baseline, participants with cancer worked significantly more hours than the general population group (Table 2). Twelve months after their diagnosis of colorectal cancer, 50 (27%) participants had stopped working (either temporarily or permanently) compared with 53 (8%) participants in a comparable 12 month period in the general population group (Table 2). Having colorectal cancer was the sole reason stated in 71% of participants who stopped work in the cancer group. A further 45 (32%) participants with colorectal cancer switched from full- to part-time work compared with 28 (6%) in the general population group. A total of 29 participants (20%) with colorectal cancer reported they had different tasks or responsibilities in their workplace since being diagnosed with cancer.

**Table 2:** **Employment changes over 12 months in the colorectal cancer and general population groups**

	Colorectal cancer		General population	*p*
	N	n (%)^1^	N = 717	
Hours worked, mean (SD)				
- at Baseline	n = 217	42.4 (15.9)	38.6 (14.4)	<0.01^2^
- at 12 months in those working	n = 132	38.2 (15.3)	38.7 (14.3)	0.73^2^
Changes in work hours per week				
- ceased work	n = 187	50 (27%)	53 (8%)	<0.01^3^
- decreased (≥4 hours)		36 (19%)	156 (23%)	
- unchanged (< ± 4 hours)		99 (53%)	377 (55%)	
- increased (≥4 hours)		2 (1%)	101 (15%)	
Days off work^4^, median (25,75%)	n = 102	91 (14,183)	N/A	-
Full- to part-time^5^	n = 140	45 (32%)	28 (6%)	<0.01^3^
Changes in income				
- reduced by >5%	n = 92	17 (19%)	111 (24%)	<0.01^3^
- increased by >5%		3 (3%)	189 (42%)	
- unchanged or changed by up to ±5%		72 (78%)	156 (34%)	
Occupation changed	n = 147	15 (10%)	43 (7%)	0.12^3^
Employer changed	n = 147	11 (8%)	30 (5%)	0.19^3^
Workplace responsibilities changed	n = 145	29 (20%)	N/A	-

^1^unless otherwise indicated.

^2^t-tests.

^3^chi-squared test.

^4^due to cancer.

^5^full time is defined as working ≥32 hours per week, part time is <32 hours per week.

### Employment among the cancer group

In the cancer group, similar proportions of men and women ceased work, reduced their work hours, or maintained their work hours from baseline to 12 months. At six months, paid sick leave accounted for 51% of income support during previous time away from work due to cancer, 24% was taken as annual leave, 6% as long-service leave, 18% as income protection insurance and the remainder as ‘other’ type of payment. Respondents reported various workplace arrangements to accommodate their absence including: employing a temporary staff member (36%), existing staff worked longer (33%), working from home (0.5%) and other (5%). No participant reported working longer days to make up for any time off.

### Factors associated with stopping work at 12 months

Individuals with colorectal cancer were more likely to stop work at 12 months if they were older, had lower body mass index (BMI), and/or lower physical well-being (Table 3). Cancer-specific factors (e.g., cancer site, stage, grade, surgical complications, length of hospital stay) or occupational factors were not associated with stopping work at 12 months (not shown). Individuals of the same age and gender from the general population were more likely to stop work if they were: older, did not study a trade or further education after high school, shorter tenure with employer, did not have a permanent work contract or were in a middle-income bracket.

**Table 3:** **Correlates of work cessation at 12 months (significant variables in the models shown only)**

	General population				Colorectal Cancer			
	Univariable Model n = 717		Multivariable Model n = 519		Univariable Model n = 239		Multivariable Model n = 176	
	OR	95% CI	OR	95% CI	OR	95% CI	OR	95% CI
Age^1^	1.16***	(1.09,1.24)	1.15***	(1.06,1.25)	1.08*	(1.00,1.16)	1.08*	(1.00,1.16)
Gender								
- male	1.00	-			1.00	-		
- female	2.26**	(1.29,3.97)			0.92	(0.46,1.81)		
BMI^1^	1.02	(0.96,1.08)			0.92*	(0.84,0.99)	0.91*	(0.84,0.99)
Education								
- junior/high school	1.00	-	1.00	-	1.00	-		
- trade/vocational	0.50*	(0.26,0.96)	0.30*	(0.12,0.76)	0.76	(0.28,2.07)		
- university	0.38*	(0.18,0.81)	0.32*	(0.12,0.88)	0.28*	(0.09,0.86)		
Child/ren in household								
- no	1.00	-			1.00	-		
- yes	0.09*	(0.01,0.65)			0.37	(0.10,1.30)		
People in household								
- 1	1.00	-			1.00	-		
- 2	0.95	(0.48,1.87)			1.05	(0.34,3.23)		
- 3	0.17**	(0.05,0.59)			0.65	(0.16,2.60)		
- 4 or more	0.14**	(0.04,0.52)			0.34	(0.08,1.50)		
Comorbidities								
- none	1.00	-			1.00	-		
- 1	2.06*	(1.06,3.99)			0.65	(0.27,1.55)		
- 2	2.76*	(1.21,6.28)			1.26	(0.47,3.38)		
- 3 or more	1.20	(0.27,5.44)			2.14	(0.81,5.66)		
Occupation group								
- professional/managerial	1.00	-			1.00	-		
- skilled services	1.93	(0.92,4.06)			3.09*	(1.08,8.82)		
- skilled trades	2.39*	(1.20,4.75)			2.88	(0.96,8.66)		
Employer size								
- <20 staff	1.00	-			1.00	-		
- 20 to 100 staff	0.62	(0.29,1.32)			2.10	(0.98,4.53)		
- >100 staff	0.28**	(0.11,0.70)			1.57	(0.52,4.77)		
- 1 (self-employed)	1.47	(0.71,3.07)			1.12	(0.11,10.82)		
Time at employer^1^ (years)	0.94***	(0.90,0.97)	0.94*	(0.89,0.99)	1.02	(0.99,1.04)		
Work contract								
- permanent	1.00	-	1.00	-	1.00	-		
- other	4.28***	(2.18,8.39)	4.57***	(2.05,10.16)	1.89	(0.85,4.24)		
Work autonomy^2^	0.88	(0.72,1.08)			0.47**	(0.28,0.77)		
Household income								
- > $104,000	1.00	-	1.00	-	1.00	-		
- < $78,000	5.92***	(2.56,13.67)	1.40	(0.52,3.82)	2.20	(0.96,5.02)		
- $78,000 to $104,000	6.19***	(2.37,16.20)	4.13**	(1.42,12.03)	1.15	(0.43,3.04)		
Income group								
- $36,000 to $78,000	1.00	-			1.00	-		
- > $78,000	0.25***	(0.12,0.52)			1.25	(0.48,3.27)		
- < $36,000	0.09***	(0.03,0.31)			0.90	(0.32,2.59)		
Energy^3^								
- none/little of the time	1.00	-			1.00	-		
- some/good bit of the time	0.46	(0.21,0.98)			0.48	(0.21,1.10)		
- most/all of the time	0.57	(0.26,1.21)			0.18***	(0.07,0.45)		
Mental Component Score^4^	0.99	(0.96,1.03)			0.98	(0.95,1.01)		
Physical Component Score^4^	0.96***	(0.93,0.98)			0.92***	(0.89,0.95)	0.93***^5^	(0.90,0.97)

^1^centred over mean.

^2^continuous variable, centred over mean, the higher the number the more flexibility the person has regarding their work hours, tasks performed, taking breaks etc.

^3^from the SF-12 scale asking respondents ‘did you have a lot of energy?’ in the past 4 weeks.

^4^continuous variable, centred over mean, from the SF-12 scale, higher scores reflect higher quality of life.

^5^multiple imputation of missing values.

^*^P < 0.05, **P < 0.01, ***P < 0.001.

### Factors associated with time to work resumption among cancer group

Participants with cancer who returned to work took a median of 91 days off work (25–75 percentiles: 14–183 days). Factors associated with delayed work re-entry after cancer included not being university-educated, poorer perceived financial status, working for a employer with more than 20 employees in a non-professional or non-managerial role, longer hospital stay, and chemotherapy (currently undergoing or treatment completed) (Table 4).

**Table 4:** **Factors associated with time to work resumption** ^**1**^ **among participants with colorectal cancer (significant variables shown only)**

	Univariable n = 195		Multivariable n = 186	
	HR	95% CI	HR	95% CI
Education				
- junior/high school	1.00	-	1.00	-
- trade/vocational	0.97	(0.56,1.68)	1.11	(0.62,1.99)
- university	1.91**	(1.30,2.81)	1.76*	(1.09,2.83)
Perceived prosperity				
- poor/just getting along	1.00	-	1.00	-
- reasonably comfortable	2.04**	(1.25,3.32)	1.80*^2^	(1.05,3.09)
- very comfortable/prosperous	2.84***	(1.55,5.22)	1.89^2^	(0.93,3.87)
Employer size				
- 20 to 100 staff	1.00	-	1.00	-
- <20 staff	1.46	(1.00,2.14)	1.66*	(1.09,2.53)
- >100 staff	1.80*	(1.09,2.96)	1.47	(0.83,2.60)
Occupation group				
- skilled services	1.00	-	1.00	-
- skilled trades	0.77	(0.50,1.20)	1.05	(0.65,1.68)
- professional/managerial	1.85**	(1.24,2.76)	1.60*	(1.02,2.52)
Stoma fitted				
- no	1.00	-		
- yes	0.50**	(0.33,0.76)		
Surgery complications				
- no	1.00	-		
- yes	0.57*	(0.35,0.92)		
Hospital length of stay^3^(days)	0.94**	(0.90,0.98)	0.93**	(0.89,0.97)
Chemotherapy				
- none	1.00	-	1.00	-
- currently undergoing	0.35***	(0.23,0.53)	0.29***	(0.19,0.45)
- treatment completed	0.36***	(0.21,0.61)	0.32***	(0.18,0.57)
Radiotherapy				
- none	1.00	-		
- any	0.47*	(0.26,0.85)		
Energy^4^				
- none/little of the time	1.00	-		
- some/good bit of the time	2.16**	(1.32,3.51)		
- most/all of the time	2.28**	(1.36,3.80)		

^1^study period ended at 12 months after diagnosis, therefore the time to work resumption data from those temporarily retired (still planning to return to work) at 12 months were censored at the end of the study period.

^2^multiple imputation of missing values.

^3^centred over mean.

^4^from the SF-12 scale asking respondents ‘did you have a lot of energy?’ in the past 4 weeks; HR (hazard ratio) <1 means lower hazard of work re-entry, ie. longer time off-work, controlling for the other variables; HR > 1 associated with shorter time off-work, controlling for the other variables.

^*^P < 0.05, **P < 0.01, ***P < 0.001.

## Discussion and conclusions

This is the first study to assess the work outcomes of men and women diagnosed with colorectal cancer during middle adulthood (45–64 years). During this pre-retirement phase, individuals with cancer were three and a half times more likely to have stopped work compared with an age and gender-matched group from the general population. This finding should be tempered by a proportion of those individuals who do not want to return to their workplace. Of those who returned, 20% reported having different work tasks and responsibilities. Only older age (but still below the retirement age of 65 years), poorer self-reported physical health and lower BMI (perhaps linked with poorer physical health) were associated with stopping work at 12 months for those with colorectal cancer however a range of occupational, socio-demographic and clinical factors were linked with a more timely work re-entry.

Our research indicates that while many individuals were able to return to work by 12 months (a few even working through their treatment regimes) many changes occurred and these changes were similar across genders. Job tasks, employer accommodations and reduced work hours were examples of work-related changes, and 27% of workers ceased work following their colorectal cancer diagnosis. A further 32% of participants with colorectal cancer switched to part-time work 12 months after diagnosis. Workplace arrangements were made in 78% of the participant group to accommodate interruptions to receive treatment. The implications of these events suggest that employers will need to be prepared for more flexible arrangements as employees require time off work. The model by Steiner *et al.* that advocates a wider social approach to examining work outcomes for cancer survivors, integrated with patient, socio-demographic factors, is indeed highly relevant [19]. This is due to our findings showing significant factors associated with work return crossing all three of these domains.

Four previous studies have specifically focused on individuals with colorectal cancer and employment participation outcomes [5, 6, 9, 21]. Other studies involving individuals with colorectal cancer assess different outcomes such as financial burden [22–25], perceived work ability [4]. Our study avoided some of the limitations of prior studies, namely, the use of cross-sectional designs [10], the use of an indirect measurement of return to work [21], the lack of a general population group [6, 10] and the associated inability to isolate the natural effects of ageing, retirement choice and labor force changes. Our findings agreed with the results of these studies in terms of chemotherapy, older age, advanced stage and lower socio-economic indicators being impediments to work participation. However, two studies [6, 21] found higher rates of colorectal cancer survivors who were not working at 12 months (range 33-40%) while another was similar to our results (17%) [5].

The strengths of our study include a population-based recruitment approach, the use of validated instruments, the inclusion of a non-cancer general population group (HILDA) and utilising both a prospective and retrospective design. We chose colorectal cancer because this cancer type is the most common to both men and women. Telephone interviews were undertaken in order to reach a wide geographical region and minimise participant burden, the latter being important in a working population that may require out-of-hours contact. We also included many items covering the context of people’s workplaces that have rarely been studied in the relationship between cancer and employment.

Our findings present a starting point from which to understand the nature of colorectal cancer upon work, the impact and the adaptations patients and employers make when facing this disease. The implications suggest that employers will need to be prepared for more flexible arrangements as employees require time off work. Employers may need to plan for an absence of around three months with many taking up to six months off and others even longer. Longer time off is necessary for those in blue-collar or physical occupations. Clear discussions between patients and employers on capacity to work and each party’s expectations will facilitate this planning [26]. More broadly, it is critical that the Government is prepared to address issues around the impending loss of skilled people from the workforce with chronic illnesses. Australia’s ageing population will reduce productivity during the next few decades with the ratio of adults not in employment to those in employment continuing to rise. Yet it appears that the Government is slow to make decisions that meet the challenges of an ageing population [27]. Rehabilitation and retraining programs to assist patients return to work as part of broader policies to increase work participation after acute illness or injury should be more attractive than increasing welfare payments or taxes. Rehabilitation and retraining programs are likely to create specific, sustainable and positive work retention measures and avoid incentivizing long-term work departure with welfare support.

Our study has a number of limitations. Our response rate was low. Possible reasons include the target group was too ill at the recruitment time or too time-poor with little capacity to join a research project. Many participants completed their interviews during work time or while receiving intravenous chemotherapy in their outpatient clinic appointments. However, in terms of their health, in general the non-participants had similar clinical presentations than participants. Another limitation was we did not ask whether individuals had a cancer recurrence during the study period and whether they had told their employer about their diagnosis. Finally, recent qualitative work has raised questions of personality and motivational factors being strong drivers for individuals in the return to their workplace after illness [27], which our study did not assess.

In conclusion, for middle-aged working men and women diagnosed with colorectal cancer, one in four were not working at 12 months after diagnosis compared with one in ten without cancer. Individuals with no tertiary education, who were undertaking chemotherapy and/or in large workplaces could be targeted for specific assistance for faster return to work after colorectal cancer.

## Electronic supplementary material

Additional file 1: Table S1: Descriptive analyses between the participants and non-participants (colorectal cancer group). **Table S2.** Descriptive analyses of baseline characteristics for completers versus non-completers (colorectal cancer group). (PDF 620 KB)

## Abbreviations

ABS: Australian Bureau of Statistics
AJCC: American Joint Committee of Cancer
BMI: Body mass index
HILDA: Household, income and labour dynamics in Australia
HRQoL: Health-related quality of life
QIMR: Queensland Institute of Medical Research.
