# Supplementary material for: The return to work experiences of middle-aged Australian workers diagnosed with colorectal cancer: a matched cohort study
Source: BMC Public Health. 2014 Sep 17;14:963. doi: 10.1186/1471-2458-14-963 (PMC4190428; doi:10.1186/1471-2458-14-963)
Supplement: Supplementary file 1 — Additional file 1: Table S1: Descriptive analyses between the participants and non-participants (colorectal cancer group). Table S2. Descriptive analyses of baseline characteristics for completers versus non-completers (colorectal cancer group). (PDF 620 KB) [file 12889_2014_7098_MOESM1_ESM.pdf]

Additional file 1

Table 1. Descriptive analyses between the participants and non-participants (colorectal cancer group)

Variables			Participated in WACS			*p*-value
			no<br>n=859	yes<br>n=239	total<br>n=1098	
Status	eligible and consented	n	0	239	239	-
		%	0.0	21.8	21.8	
	no consent (by patient)	n	466	0	466	
		%	42.4	0.0	42.4	
	no consent (by doctor)	n	393	0	393	
		%	35.8	0.0	35.8	
	missing	n	0	0	0	-
Age	years in 2010	median (IQR)	58.0 (9.0)	57.0 (10.0)	58 .0 (10.0)	0.017[†]
		min-max	45-65	45-65	45-65	
	missing	n	0	0	0	-
Gender	male	n	500	159	659	0.020[◊]
		%	58.2	66.5	60.0	
	female	n	359	80	439	
		%	41.8	33.5	40.0	
	missing	n	0	0	0	-
Area1	capital city	n	345	86	431	0.715[◊]
		%	40.2	36.0	39.3	
	other metropolitan	n	143	39	182	
		%	16.7	16.3	16.6	
	large rural centre	n	116	37	153	
		%	13.5	15.5	14.0	
	small rural centre	n	66	15	81	
		%	7.7	6.3	7.4	
	other rural centre	n	144	50	194	
		%	16.8	20.9	17.7	
	remote centre	n	21	6	27	
		%	2.5	2.5	2.5	
	other remote area	n	23	6	29	
		%	2.7	2.5	2.6	
	missing	n	1	0	1	-
Area2	major city	n	464	117	581	0.352[◊]
		%	54.1	49.0	53.0	
	inner regional	n	186	66	252	
		%	21.7	27.6	23.0	
	outer regional	n	158	44	202	
		%	18.4	18.4	18.4	
	remote	n	28	8	36	
		%	3.3	3.4	3.3	
	very remote	n	22	4	26	
		%	2.6	1.7	2.4	
	missing	n	1	0	1	-
Excision or biopsy	excision	n	444	143	587	0.130[◊]
		%	54.6	60.1	55.8	
	biopsy	n	370	95	465	
		%	45.5	39.9	44.2	
	missing	n	45	1	46	-
Cancer site	colon	n	412	105	517	0.470[◊]
		%	48.6	44.1	47.6	
	rectum	n	292	90	382	
		%	34.4	37.8	35.2	

Variables			Participated in WACS no n=859	Participated in WACS yes n=239	Participated in WACS total n=1098	*p*-value
	unknown	n	144	43	187	
		%	17.0	18.1	17.2	
	missing	n	11	1	12	-
Histology	adenocarcinoma	n	790	225	1,015	0.728$^{\diamond}$
		%	93.9	94.5	94.1	
	other	n	51	13	64	
		%	6.1	5.5	5.9	
	missing	n	18	1	19	-
Grade	well diff./low	n	84	19	103	0.325$^{\diamond}$
		%	12.7	9.5	11.9	
	mod. diff./intermed.	n	464	151	615	
		%	70.0	75.1	71.2	
	poorly diff./high	n	115	31	146	
		%	17.4	15.4	16.9	
	missing	n	196	38	234	-
AJCC staging	0	n	7	0	7	0.091$^{\diamond}$
		%	1.8	0.0	1.3	
	I	n	110	46	156	
		%	28.0	33.8	29.5	
	IIA	n	93	33	126	
		%	23.7	24.3	23.8	
	IIB	n	18	7	25	
		%	4.6	5.2	4.7	
	IIIA	n	19	5	24	
		%	4.8	3.7	4.5	
	IIIB	n	72	21	93	
		%	18.3	15.4	17.6	
	IIIC	n	41	21	62	
		%	10.4	15.4	11.7	
	IV	n	33	3	36	
		%	8.4	2.2	6.8	
	missing	n	466	103	569	-
Tumour size / invaded nearby tissue	no signs of tumour	n	1	0	1	0.463$^{\Delta}$
		%	0.2	0.0	0.2	
	size 1	n	57	24	81	
		%	13.9	17.7	14.8	
	size 2	n	77	28	105	
		%	18.7	20.6	19.2	
	size 3	n	204	67	271	
		%	49.6	49.3	49.5	
	size 4	n	64	17	81	
		%	15.6	12.5	14.8	
	carcinoma in situ	n	8	0	8	
		%	2.0	0.0	1.5	
	missing	n	448	103	551	-
Regional lymph nodes	tumor cells absent fr	n	209	83	292	0.386$^{\Delta}$
		%	54.3	62.4	56.4	
	reg/lymph node metast	n	101	28	129	
		%	26.2	21.1	24.9	
	spread to an extent b	n	73	22	95	
		%	19.0	16.5	18.3	
	cannot be evaluated	n	2	0	2	
		%	0.5	0.0	0.4	
	missing	n	474	106	580	-

Variables			Participated in WACS no n=859	yes n=239	total n=1098	*p*-value
Spread of cancer from one body part to another	no distant metastasis	n	57	28	85	0.045◊
		%	31.5	40.6	34.0	
	metastasis to distant	n	32	4	36	
		%	17.7	5.8	14.4	
	cannot be evaluated	n	92	37	129	
		%	50.8	53.6	51.6	
	missing	n	678	170	848	-
Dukes staging	invasion into but not	n	37	25	62	0.005◊
		%	10.6	20.0	13.1	
	invasion through the	n	151	51	202	
		%	43.1	40.8	42.5	
	involvement of lymph	n	128	46	174	
		%	36.6	36.8	36.6	
	widespread metastases	n	34	3	37	
		%	9.7	2.4	7.8	
	missing	n	509	114	623	-

† *t-test with equal variances used*

◊ *Pearson's chi-squared test used*

Δ *Fisher's exact test used*

Table 2. Descriptive analyses of baseline characteristics for completers versus non-completers (colorectal cancer group)

Variables	Value labels, etc.		Phone interview, no. completed *n*	<two n=57	two n=182	total n=239	*p*	Postal survey, no. completed *n*	<two n=77	two n=162	total n=239	*p*
Age in 2010	median (IQR) years		239	58 (9)	57 (9)	58 (9)	0.575	239	58 (9)	56.5 (9)	57 (9)	0.957
	range (years)			45-64	45-65	45-65			45-64	46-65	45-65	
Gender	male		239	43	117	160	0.118	239	57	103	160	0.109
		%		75.4	64.3	67.0			74.0	63.6	67.0	
	female			14	65	79			20	59	79	
		%		24.6	35.7	33.1			26.0	36.4	33.1	
Country of birth	Australia		222	34	146	180	0.895	222	56	124	180	0.800
		%		79.07	81.56	81.08			82.4	80.5	81.1	
	English speaking			5	20	25			8	17	25	
		%		11.63	11.17	11.26			11.8	11.0	11.3	
	other			4	13	17			4	13	17	
		%		9.3	7.26	7.66			5.9	8.4	7.7	
First Peoples	no		222	42	177	219	0.478	222	67.0	152.0	219.0	1.000
		%		97.67	98.88	98.65			98.5	98.7	98.7	
	yes			1	2	3			1.0	2.0	3.0	
		%		2.33	1.12	1.35			1.5	1.3	1.4	
ARIA	major city		239	29	88	117	0.738	239	34	83	117	0.026
		%		50.9	48.4	49.0			44.2	51.2	49.0	
	inner regional			13	53	66			16	50	66	
		%		22.8	29.1	27.6			20.8	30.9	27.6	
	outer regional			13	31	44			21	23	44	
		%		22.8	17.0	18.4			27.3	14.2	18.4	
	remote			1	7	8			3	5	8	
		%		1.8	3.9	3.4			3.9	3.1	3.4	
	very remote			1	3	4			3	1	4	
		%		1.8	1.7	1.7			3.9	0.6	1.7	
Area1	capital city		239	20	66	86	0.533	239	28	58	86	0.266

Variables	Value labels, etc.	Phone interview, no. completed					Postal survey, no. completed				
		n	<two n=57	two n=182	total n=239	*p*	*n*	<two n=77	two n=162	total n=239	*p*
	%		35.1	36.3	36.0			36.36	35.8	35.98	
	other metropolitan		13	26	39			11	28	39	
	%		22.8	14.3	16.3			14.29	17.28	16.32	
	large rural centre		9	28	37			13	24	37	
	%		15.8	15.4	15.5			16.88	14.81	15.48	
	small rural centre		1	14	15			4	11	15	
	%		1.8	7.7	6.3			5.19	6.79	6.28	
	other rural centre		11	39	50			14	36	50	
	%		19.3	21.4	20.9			18.18	22.22	20.92	
	remote centre		2	4	6			5	1	6	
	%		3.5	2.2	2.5			6.49	0.62	2.51	
	other remote area		1	5	6			2	4	6	
	%		1.8	2.8	2.5			2.6	2.47	2.51	
Marital status	single	222	6	16	22	0.506	222	8	14	22	0.231
	%		13.95	8.94	9.91			11.76	9.09	9.91	
	widowed		0	2	2			1	1	2	
	%		0	1.12	0.9			1.47	0.65	0.9	
	divorced/separated		5	14	19			9	10	19	
	%		11.63	7.82	8.56			13.24	6.49	8.56	
	married/partnered		32	147	179			50	129	179	
	%		74.42	82.12	80.63			73.53	83.77	80.63	
Education	no formal schooling	221	0	1	1	0.904	221	0	1	1	0.853
	%		0	0.56	0.45			0	0.65	0.45	
	primary school		3	14	17			6	11	17	
	%		6.98	7.87	7.69			8.96	7.14	7.69	
	junior high school		16	74	90			27	63	90	
	%		37.21	41.57	40.72			40.3	40.91	40.72	
	senior high school		7	29	36			11	25	36	
	%		16.28	16.29	16.29			16.42	16.23	16.29	
	trade/TAFE/diploma		5	24	29			11	18	29	
	%		11.63	13.48	13.12			16.42	11.69	13.12	
	university		12	36	48			12	36	48	
	%		27.91	20.22	21.72			17.91	23.38	21.72	
Excision or biopsy	excision	238	37	106	143	0.393	238	44	99	143	0.522
	%		64.9	58.6	60.1			57.14	61.49	60.08	
	biopsy		20	75	95			33	62	95	
	%		35.1	41.4	39.9			42.86	38.51	39.92	
Cancer site	colon	238	25	80	105	0.594	238	36	69	105	0.847
	%		43.9	44.2	44.1			46.75	42.86	44.12	
	rectum		24	66	90			28	62	90	
	%		42.1	36.5	37.8			36.36	38.51	37.82	
	unknown		8	35	43			13	30	43	
	%		14.0	19.3	18.1			16.88	18.63	18.07	
Histology	adenocarcinoma	238	56	169	225	0.199	238	74	151	225	0.556
	%		98.3	93.4	94.5			96.1	93.79	94.54	
	other		1	12	13			3	10	13	
	%		1.8	6.6	5.5			3.9	6.21	5.46	
Grade	well diff./low	201	5	14	19	0.296	201	4	15	19	0.285
	%		10.4	9.2	9.5			6.25	10.95	9.45	
	mod. diff./intermed.		39	112	151			47	104	151	
	%		81.3	73.2	75.1			73.44	75.91	75.12	
	poorly diff./high		4	27	31			13	18	31	
	%		8.3	17.7	15.4			20.31	13.14	15.42	
AJCC staging	0	136	-	-	-	0.575	136	-	-	-	0.087

Variables	Value labels, etc.	Phone interview, no. completed					Postal survey, no. completed				
		n	<two n=57	two n=182	total n=239	*p*	*n*	<two n=77	two n=162	total n=239	*p*
	%		-	-	-			-	-	-	
	I		12	34	46			15	31	46	
	%		34.3	33.7	33.8			34.88	33.33	33.82	
	IIA		8	25	33			7	26	33	
	%		22.9	24.8	24.3			16.28	27.96	24.26	
	IIB		0	7	7			0	7	7	
	%		0.0	6.9	5.2			0	7.53	5.15	
	IIIA		2	3	5			3	2	5	
	%		5.7	3.0	3.7			6.98	2.15	3.68	
	IIIB		6	15	21			6	15	21	
	%		17.1	14.9	15.4			13.95	16.13	15.44	
	IIIC		7	14	21			11	10	21	
	%		20.0	13.9	15.4			25.58	10.75	15.44	
	IV		0	3	3			1	2	3	
	%		0.0	3.0	2.2			2.33	2.15	2.21	
No. of people in household	median (IQR)	222	2 (1.0)	2 (1.0)	2 (1.0)	0.829	222	2 (1.0)	2 (1.0)	2 (1.0)	0.829
	range		1-6	1-6	1-6			1-6	1-6	1-6	
No. of <15yo in household	median (IQR)	221	0 (0.0)	0 (0.0)	0 (0.0)	0.419	221	0 (0.0)	0 (0.0)	0 (0.0)	0.941
	range		0-2	0-3	0-3			0-2	0-3	0-3	
Annual household income gross AUD2010-11	less than $ 10399	215	0	2	2	0.102	215	1	1	2	0.174
	%		0	1.14	0.93			1.54	0.67	0.93	
	$ 10400 - $ 36399		3	11	14			4	10	14	
	%		7.5	6.29	6.51			6.15	6.67	6.51	
	$ 36400 - $ 77999		7	47	54			11	43	54	
	%		17.5	26.86	25.12			16.92	28.67	25.12	
	$ 78000 - $103999		6	41	47			18	29	47	
	%		15	23.43	21.86			27.69	19.33	21.86	
	$104000 - above		15	61	76			21	55	76	
	%		37.5	34.86	35.35			32.31	36.67	35.35	
	refused to answer		9	13	22			10	12	22	
	%		22.5	7.43	10.23			15.38	8	10.23	
Long-term health condition: arthritis	no	222	38	144	182	0.225	222	57	125	182	0.635
	%		88.37	80.45	81.98			83.82	81.17	81.98	
	yes		5	35	40			11	29	40	
	%		11.63	19.55	18.02			16.18	18.83	18.02	
Long-term health condition: asthma	no		39	163	202	1.000		63	139	202	0.567
	%		90.7	91.06	90.99			92.65	90.26	90.99	
	yes		4	16	20			5	15	20	
	%		9.3	8.94	9.01			7.35	9.74	9.01	
Long-term health condition: cancer	no		37	156	193	0.847		60	133	193	0.703
	%		86.05	87.15	86.94			88.24	86.36	86.94	
	yes		6	23	29			8	21	29	
	%		13.95	12.85	13.06			11.76	13.64	13.06	
Long-term h/cond: chronic bronchitis/ emphysema	no		40	170	210	1.000		61	149	210	0.073
	%		95.24	94.97	95.02			91.04	96.75	95.02	
	yes		2	9	11			6	5	11	
	%		4.76	5.03	4.98			8.96	3.25	4.98	
Long-term h/cond: diabetes (childhood onset)	no		42	178	220	1.000		66	154	220	0.129
	%		100	99.44	99.55			98.51	100	99.55	
	yes		0	1	1			1	0	1	
	%		0	0.56	0.45			1.49	0	0.45	
Long-term health condition:	no		38	159	197	1.000		57	140	197	0.200
	%		90.48	88.83	89.14			85.07	90.91	89.14	
	yes		4	20	24			10	14	24	

Variables	Value labels, etc.		Phone interview, no. completed					Postal survey, no. completed				
				<two	two	total			<two	two	total	
			n	n=57	n=182	n=239	*p*	*n*	n=77	n=162	n=239	*p*
type2 diabetes (adult onset)		%		9.52	11.17	10.86			14.93	9.09	10.86	
Long-term health condition: depression/ anxiety	no			38	163	201	1.000		58	143	201	0.134
		%		90.48	91.06	90.95			86.57	92.86	90.95	
	yes			4	16	20			9	11	20	
		%		9.52	8.94	9.05			13.43	7.14	9.05	
Long-term health condition: heart/ coronary disease	no			40	166	206	0.742		60	146	206	0.154
		%		95.24	92.74	93.21			89.55	94.81	93.21	
	yes			2	13	15			7	8	15	
		%		4.76	7.26	6.79			10.45	5.19	6.79	
Long-term health condition: high blood pressure	no			32	133	165	0.800		44	121	165	0.043
		%		76.19	74.3	74.66			65.67	78.57	74.66	
	yes			10	46	56			23	33	56	
		%		23.81	25.7	25.34			34.33	21.43	25.34	
Long-term health condition: diverticulitis	no			38	169	207	0.311		62	145	207	0.650
		%		90.48	94.41	93.67			92.54	94.16	93.67	
	yes			4	10	14			5	9	14	
		%		9.52	5.59	6.33			7.46	5.84	6.33	
Long-term health condition: inlf. bowel disease	no			40	175	215	0.320		63	152	215	0.070
		%		95.24	97.77	97.29			94.03	98.7	97.29	
	yes			2	4	6			4	2	6	
		%		4.76	2.23	2.71			5.97	1.3	2.71	
Long-term health condition: other circ. condition	no			42	168	210	0.350		66	144	210	0.441
		%		100	96	96.77			98.51	96	96.77	
	yes			0	7	7			1	6	7	
		%		0	4	3.23			1.49	4	3.23	
Dukes stage	invasion into bowel wall		125	7	18	25	0.418	125	7	18	25	0.241
		%		21.88	19.35	20			17.07	21.43	20	
	invasion through wall			10	41	51			13	38	51	
		%		31.25	44.09	40.8			31.71	45.24	40.8	
	inv. of lymph nodes			15	31	46			20	26	46	
		%		46.88	33.33	36.8			48.78	30.95	36.8	
	widespread metastases			0	3	3			1	2	3	
		%		0	3.23	2.4			2.44	2.38	2.4	
Received chemotherapy*	no		228	26	79	105	0.111	228	34	71	105	0.612
		%		56.52	43.41	46.05			48.57	44.94	46.05	
	yes			20	103	123			36	87	123	
		%		43.48	56.59	53.95			51.43	55.06	53.95	
Received radiotherapy*	no		226	40	149	189	0.146	226	61	128	189	0.339
		%		90.91	81.87	83.63			87.14	82.05	83.63	
	yes			4	33	37			9	28	37	
		%		9.09	18.13	16.37			12.86	17.95	16.37	
Laparoscopic surgery	no		214	12	73	85	0.128	214	28	57	85	0.507
		%		29.27	42.2	39.72			43.08	38.26	39.72	
	yes			29	100	129			37	92	129	
		%		70.73	57.8	60.28			56.92	61.74	60.28	
Were you fitted with a stoma?	no		214	27	123	150	0.905	214	43	107	150	0.625
		%		67.5	70.69	70.09			69.35	70.39	70.09	
	yes, fitted with colo			11	41	52			17	35	52	
		%		27.5	23.56	24.3			27.42	23.03	24.3	
	yes, fitted with ileo			2	10	12			2	10	12	
		%		5	5.75	5.61			3.23	6.58	5.61	
Stoma temp. or perm. fitted	temporary stoma still		61	5	25	30	0.765	61	8	22	30	1.000
		%		41.67	51.02	49.18			47.06	50	49.18	

Variables	Value labels, etc.	Phone interview, no. completed					Postal survey, no. completed				
			<two	two	total			<two	two	total	
		n	n=57	n=182	n=239	*p*	*n*	n=77	n=162	n=239	*p*
	temporary stoma no lo		4	14	18			5	13	18	
	%		33.33	28.57	29.51			29.41	29.55	29.51	
	permanent stoma		3	10	13			4	9	13	
	%		25	20.41	21.31			23.53	20.45	21.31	

** - over the 12 months study period*
